# Evaluation of scientific quality of YouTube video content related to orthodontic pain management

**DOI:** 10.1590/2177-6709.28.5.e232386.oar

**Published:** 2023-11-03

**Authors:** Pinky SINGH, Kaleem FATIMA, Ganesh CHAUDHARY, Prabhat Kumar CHAUDHARI

**Affiliations:** 1 Bharatpur Hospital, Department of Orthodontics and Dentofacial Orthopedics (Bharatpur-10, Chitwan, 44200, Nepal).; 2 Maulana Azad Institute of Dental Sciences, Department of Orthodontic and Dentofacial Orthopedics (New Delhi, 110002, India).; 3 Bharatpur Hospital, Department of Oral and Maxillofacial Surgery (Bharatpur-10 Chitwan, 44200, Nepal).; 4 All India Institute of Medical Sciences, Centre for Dental Education and Research, Division of Orthodontics and Dentofacial Deformities (New Delhi, 110029, India).

**Keywords:** Internet, Consumer health information, Orthodontists, Pain management

## Abstract

**Introduction:**

With the abundant use of the internet, patients undergoing or interested in orthodontic treatment try to use it to obtain information on pain during treatment. However, YouTube™ is unregulated and may potentially contain inaccurate information.

**Objectives:**

Thus, this study aimed to evaluate the scientific quality of the videos on YouTube™ related to orthodontic pain management.

**Methods:**

A total of 62 videos related to orthodontic pain management were included in the study. All videos were evaluated by two experienced orthodontists. The video uploader, content, length, upload date, time since upload, number of views, comments, likes, dislikes, Interaction index, and Viewing rate of the videos were recorded and evaluated. The videos were scored using the Quality Criteria for Consumer Health Information (DISCERN), Global Quality Scale (GQS), and Audio-Visual Quality (AVQ), and divided into two groups: Doctors and Non-doctors.

**Results:**

The mean DISCERN score was 2.56 ± 0.91, the GQS score was 2.56 ± 1.06, and AVQ was 2.48 ± 0.68. A statistically significant difference was found in DISCERN score of videos uploaded by Doctors compared to Non-doctors, but no statistically significant difference was found in GQS and AVQ scores between both groups (*p*> 0.05).

**Conclusions:**

The videos uploaded by Doctors were better in terms of quality and reliability, as compared to Non-doctors; and the AVQ of the videos uploaded by both groups was adequate. Despite that, both groups did not serve as a good source of information. YouTube™ cannot be considered a reliable source of information in terms of quality and reliability on videos related to orthodontic pain management.

## RESUMO

**Introdução:**

Com o uso abundante da Internet, os pacientes em tratamento ortodôntico, ou interessados em fazê-lo, tentam usá-la para obter informações sobre a dor durante o tratamento. Entretanto, o YouTube™ não é regulamentado e pode conter informações imprecisas.

**Objetivos:**

Esse estudo teve como objetivo avaliar a qualidade científica de vídeos no YouTube™ relacionados ao controle da dor ortodôntica.

**Métodos:**

No total, 62 vídeos relacionados ao controle da dor ortodôntica fora incluídos nesse estudo. Todos os vídeos foram avaliados por dois ortodontistas experientes. O responsável pela postagem do vídeo, seu conteúdo, sua duração, data de postagem, tempo decorrido desde a postagem, o número de visualizações, os comentários, os *likes*, os *deslikes*, o índice de interação e a taxa de visualização dos vídeos foram registrados e avaliados. Os vídeos foram pontuados usando os Critérios de Qualidade para Informações sobre Saúde do Consumidor (DISCERN), a Escala de Qualidade Global (GQS) e a Qualidade Audiovisual (AVQ), e divididos em dois grupos: Doutores e Não Doutores.

**Resultados:**

A pontuação DISCERN média foi de 2,56 ± 0,91, a pontuação GQS foi de 2,56 ± 1,06 e a AVQ foi de 2,48 ± 0,68. Foi encontrada uma diferença estatisticamente significativa na pontuação DISCERN dos vídeos postados por Doutores, em comparação com os Não Doutores, mas não foi encontrada diferença estatisticamente significativa nas pontuações GQS e AVQ entre os dois grupos (*p*> 0,05).

**Conclusões:**

Os vídeos postados pelos Doutores foram melhores em termos de qualidade e confiabilidade, em comparação com os Não Doutores, e o AVQ dos vídeos postados por ambos os grupos foi adequado. Apesar disso, ambos os grupos não serviram como uma boa fonte de informações. Em termos de qualidade e confiabilidade dos vídeos relativos ao manejo da dor ortodôntica, o YouTube™ não pode ser considerado uma fonte confiável de informações.

## INTRODUCTION

Traditionally, the main responsibility for health information lay with health professionals and organizations. However, with the advancement of science and technology, the use of the internet has become fundamental in our day-to-day activities. A significant population of the world has access to the worldwide web, and multiple websites provide information about modern healthcare modalities. Moreover, with the rise of social media platforms, information can now be disseminated to a wider range of people. Recent surveys report that 80% of internet users access online health information.^1^ Patients are able to access health information at a reduced cost, compared to a professional healthcare consultation. Patients not only seek medical information but also scroll for diagnosis and treatment purposes.^2^ The online media content feed is full of information and misinformation that allows patients to cross-reference information provided by the doctor, as well as gather new, additional or conflicting material.^3^

YouTube™ (www.youtube.com) is a pioneer video-sharing platform and is the second-largest search engine, next to Google. YouTube™ is available in 80 languages, reaching 95% of the Internet population. Most YouTube™ users fall in the age group of 15-35 years.^4^ Patients prefer YouTube™ because of its ease of access and social nature, providing both audio and visual media that gives a vivid understanding of the subject. Its popularity has made it a powerful tool for influencing individual decisions and promoting their well-being.^5^ ^,^ ^6^

Pain is one of the most negative effects of orthodontic treatment, and is of a major concern to parents, patients and clinicians.^7^ ^,^ ^8^ Orthodontic pain is due to changes in the blood flow following force application, leading to cascades of inflammatory reactions, releasing various chemical and neurogenic mediators that express a hyperalgesic response.^9^ ^,^ ^10^ The incidence of pain symptoms among orthodontically treated patients has been reported to be around 70% to 95%. Researchers have shown that fear of anticipated pain keeps some patients away from orthodontic treatment. One in ten orthodontic patients may discontinue treatment due to pain disorders during the early stages of treatment.^11^ Given the growing influence of the internet, it can be assumed that patients undergoing or interested in orthodontic treatment will attempt to use it to obtain information on pain during treatment. As YouTube™ is unregulated and is firmly built upon the principle of free expression, it may contain potentially inaccurate information, particularly because of anecdotal reporting and personal opinion.^12^ Thus, this study aimed to evaluate the content quality, reliability, and audio-visual quality (AVQ) of the videos related to orthodontic pain management available on YouTube.

## MATERIAL AND METHODS

### YOUTUBE™ VIDEOS SEARCH AND SELECTION

This study did not require ethics committee approval as it used publicly available data. A search was made on YouTube™ in January 2023 for videos related to orthodontic pain management, using two search terms: “orthodontic pain management” and “braces pain management”. To avoid any bias, the computer history and cookies were deleted. The videos were sorted using the ‘‘relevance’’ filter, a default option on YouTube, which uses a complex algorithm based on the number of views, upload date, rating, comments, etc. Ads displayed by YouTube™ at the beginning and end of the search results were not taken into account.

**Figure 1:**
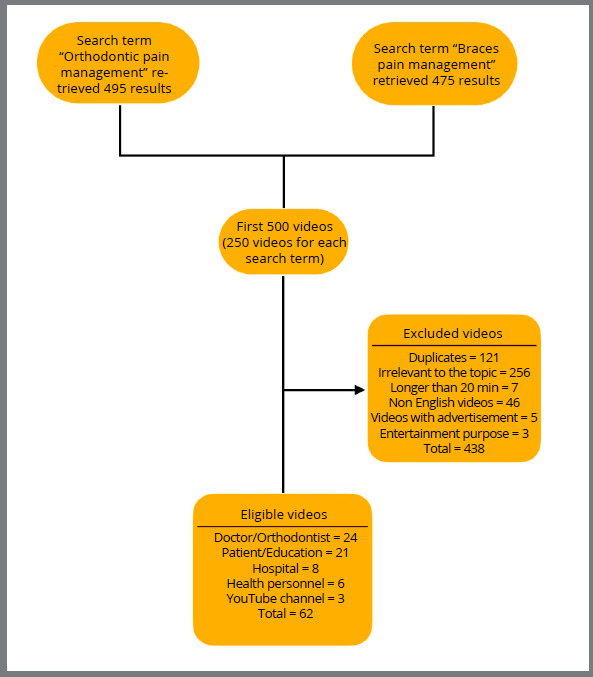
YouTube™ videos selection criteria.

Previous research has indicated that 95% of users who perform an online search on YouTube™ do not watch more than the first 60 videos in the result, and most studies utilizing YouTube™ as a search engine have used 60-200 videos.^13^ ^,^ ^14^ In the present study, the first 250 videos for each search term (i.e., a total of 500 videos) excluding YouTube™ shorts were analyzed. Since search results frequently change at different moments, two new playlists were created to add videos for each search term in the YouTube™ library, and further both were merged to create a new playlist. The exclusion criteria used were the following: (1) Duplicate videos; (2) Videos not related to orthodontic pain management; (3) Videos longer than 20 minutes; (4) Videos in languages other than English; (5) Videos uploaded for entertainment purposes; (6) Videos uploaded for the purpose of advertising a product (Fig. 1).

## ASSESSMENT OF VIDEOS

A total of 62 videos were found to be relevant for assessment (Fig 1). The videos were assessed and evaluated independently by two different investigators (PS and KF), with 6 years and 5 years of experience respectively. The video assessment and rating was approved by a Senior Professor (PKC) with more than 15 years of experience. All videos were viewed in full, and the following parameters were evaluated for each one: (i) number of views, (ii) duration (minutes), (iii) number of comments, and (iv) number of “likes” and “dislikes.” The exact number of views, likes, dislikes, and comments were calculated using freely available online software. Uploaded videos were from different sources: (i) Doctor/Orthodontist, (ii) Patient/layman, (iii) Health personnel, (iv) Clinic/hospital channel, and (v) YouTube™ channel. The criteria for video uploaders classified as Doctors included videos posted by certified Orthodontists; the Patient/layman category included videos from patients and layman; Health personnel category included videos from other health professionals (like nurses, health assistants, and dental assistants); Hospital/clinic category included videos from hospitals/clinics describing the facilities and treatments available solely to attract patients; and the YouTube™ channel category included those videos that described natural means for orthodontic pain management, such as Yoga, Meditation, etc. Furthermore, the videos were divided into Doctors and Non-doctors, grouped according to the quality of the uploader. 

The viewers interaction was calculated using the interaction index and viewing rate formulas. Video interaction was calculated through the difference in the total number of “likes” and “dislikes”, divided by the total number of views. The video viewing rate was calculated by dividing the total number of views by the number of days of the video on YouTube.^2^

$$\;Interaction\;index\;(\%)=\frac{\;Total\;no.\;of\;likes\;-\;Total\;no.\;of\;dislikes\;}{\;Total\;no.\;of\;views\;}\times 100$$

$$\;Viewing\;rate\;(\%)=\frac{\;Total\;no.\;of\;views\;}{\;Total\;no.\;of\;days\;elapsed\;}\times 100$$

**Figure 2:**
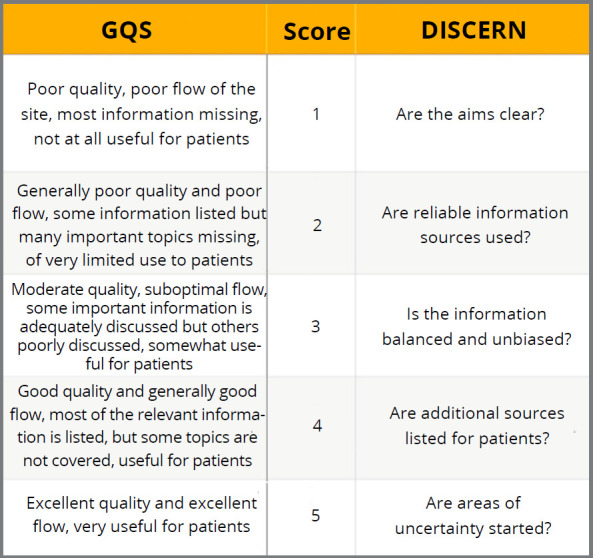
The Global Quality Scale ( GQS ) and DISCERN scoring criteria.

The AVQ of the videos was assessed according to Sorensen et al.^15^ Videos that had clear images and text, good quality graphics or effects, and had no difficulty in understanding spoken words and music were rated as ‘good’; while homemade videos, videos with regular quality and average text clarity, sentences that were difficult to understand, distracting audio or background sounds were rated as ‘moderate’; and videos containing blurry, grainy, or difficult to understand images and no audio were rated as ‘poor’. To evaluate video quality, a 5-point scale, the Global Quality Scale (GQS),was used.^16^ ^,^ ^17^ The reliability of information was assessed according to a scoring system derived from the DISCERN tool, based on 5 questions.^18^ For each question, the answer ‘‘no’’ was scored as 0 points and the answer ‘‘yes’’ was scored as 1 point. A Reliability Score (RS) was obtained by summing the total of these points. A higher score meant that the video had reliable content.^19^ (Fig 2). 

In this study, a total of 1 to 2 points on both DISCERN and GQS scales was rated as low-quality and misleading content; 3 points, as content of moderate quality; and 4 to 5 points, as useful and good quality content. Twenty videos were randomly selected using an online software (Research Randomizer, *https://www.randomizer.org/*), and examined again by both operators after two weeks, to calculate the intra-rater reliability.

## STATISTICS

Data were independently collected by the two investigators using a Microsoft Excel (Microsoft) spreadsheet, and statistical evaluations were performed using Statistical Package for the Social Sciences software (SPSS version 25; IBM Corp, SPSS Inc, Armonk, USA). Normality of the data distribution was evaluated using the Shapiro-Wilk test. The variables were expressed as mean ± standard deviation, numbers, and percentages. Chi-square tests were used for the comparison of the videos between Doctors and Non-doctors. The degree of agreement between investigators was assessed using Cohen’s Kappa coefficient (κ) score, and Intra-class correlation coefficients (ICCs) were calculated to define intra-rater reliability. Pearson correlation coefficients were calculated to evaluate possible correlations between GQS, AVQ, and RS. Statistical significance was evaluated at *p* < 0.05. 

## RESULTS

### VIDEO DEMOGRAPHICS ACCORDING TO DOCTOR AND NON-DOCTOR UPLOADER

The total number of views on the videos was 5,344,065, with a higher viewing count on the Non-doctors videos (98,873.34 ± 231,024), as compared to the Doctors videos (66,122.041 ± 109,690). The Non-doctors videos were longer in length (6.125 ± 3.85 minutes), with a higher viewing rate (7,327.2994 ± 19,386.1341), in comparison to the Doctors videos, which were shorter (3.55 ± 2.55 minutes) and had a lower viewing rate (4,847.1954 ± 7,638.2913). The videos uploaded by Doctors had a higher number of likes (1,098.54 ± 1,954.77), as compared to Non-doctors videos (1,736.32 ± 4,785.81). On the other hand, the Non-doctors videos had a higher number of dislikes (51.61 ± 131.68), as compared to the Doctors videos (20.13 ± 32.8). The videos uploaded by Doctors were better in terms of AVQ (2.708 ± 0.7506), GQS (2.896 ± 1.103) and reliability score (3.0208 ± 0.960), as compared to the Non-doctors videos (AVQ = 2.342 ± 0.6163), (GQS = 2.355 ± 0.9994), (RS = 2.2632 ± 0.7510). The demographics for the videos on the groups Doctors and Non-doctors, and for all the videos, expressed in terms of mean and standard deviation, are presented in Table 1.

**Table 1:** Video demographics for Doctor and Non-doctor uploaders.

Characteristics	Doctors (n=24) (Mean ± SD)	Non-doctors (38) (Mean ± SD)	All videos (n=62) (Mean ± SD)
Viewing counts	66,122.04167 ± 109,690.3183	98,873.34211 ± 231,024.1196	86,195.4193 ± 192,791.5225
Likes	1,098.54 ± 1,954.770	1,736.32 ± 4,785.813	1,489.44 ± 1,489.266
Dislikes	20.13 ± 32.860	51.61 ± 131.681	39.42 ± 105.658
Comments	250.96 ± 424.243	495.97 ± 1,694.970	401.13 ± 1,350.900
Video length	3.5513 ± 2.55235	6.1250 ± 3.85214	5.1287 ± 3.61308
Time elapsed	1,274.92 ± 1,038.088	1,490.74 ± 1,039.150	1,407.19 ± 1,035.631
Interaction index	1. 62875 ± 0.9809	1.5276 ± 1.0976	1.5667 ± 1.0469
Viewing rate	4,847.1954 ± 7,638.2913	7,327.2994 ± 19,386.1341	6,367.2591 ± 15,856. 8388
Audiovisual quality	2.708 ± 0.7506	2.342 ± 0.6163	2.484 ± 0.6893
Global quality Scale	2.896 ± 1.1031	2.355 ± 0.9994	2.565 ± 1.0654
Reliability score	3.0208 ± 0.9609	2.2632 ± 0.7510	2.5565 ± 0.9103

### ASSESSMENT OF VIDEOS FOR AVQ, GQS, AND RS

**Figure 3:**
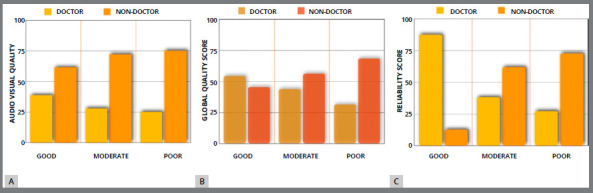
A) Percentages distribution of the Audio-visual Quality ( AVQ ) score between Doctors and Non-doctors videos. B) Percentages distribution of the Global Quality Score ( GQS ) scores between Doctors and Non-doctors videos . C) Percentages distribution of Reliability Score ( RS ) between Doctors and Non-doctors videos.

The quality of AVQ, GQS and RS was expressed in numbers and percentages. The differences regarding AVQ (*p* = 0.23) and GQS (*p*= 0.347) between the Doctors and Non-doctors videos were not significant. The RS of videos was significantly different between the groups: the Doctors’ videos had more reliability than the Non-doctors’ videos (p=0.049) (Table 2) (Fig 3). 

**Table 2:** Quality of videos in Doctors and Non-doctors groups, expressed in percentage and absolute number.

	AVQ			GQS			RS		
	Doctor	Non-doctor	Total	Doctor	Non- doctor	Total	Doctor	Non-doctor	Total
Poor quality	25% (n = 1)	75% (n = 3)	6.45% (n = 4)	31.4% (n = 11)	68.6% (n = 24)	56.45% (n = 35)	27.3% (n = 9)	72.7% (n = 24)	53.22% (n = 33)
Moderate quality	28% (n = 7)	72% (n = 18)	40.32% (n = 25)	43.8% (n = 7)	56.3% (n = 9)	25.80% (n = 16)	38.1% (n = 8)	61.9% (n = 13)	35.48% (n = 21)
Good quality	38.7% (n = 16)	61.3% (n = 17)	53.22% (n = 33)	54.5% (n = 6)	45.5% (n = 5)	18.33% (n = 11)	87.5% (n = 7)	12.5% (n = 1)	11.30% (n = 8)
Total	24	38	100 % (n = 62)	24	38	100% (n = 62)	24	38	100% (n = 62)
P- value	0.235			0.347			0.049		

AVQ = Audio-Visual Quality. GQS = Global Quality Score. RS = Reliability Score.

There was a moderate correlation between GQS and AVQ (r = 0.548, *p*< 0.01), and between RS and AVQ (r = 0.622, *p*< 0.01). There was a good correlation between GQS and RS (r = 0.879, *p*< 0.01). There was a poor correlation among GQS, RS, and AVQ with the number of views, likes, dislikes, and comments on the videos (Table 3).

**Table 3:** Correlation matrix displaying Pearson correlation coefficients amongst AVQ score, GQS, Reliability score, and video demographics.

Variables	AVQ	GQS	RS	n°. of views	n°. of likes	n°. of dislikes	n°. of comments	Video length	n°. of days elapsed
AVQ	1	0.548**	0.622**	0.162	0.152	0.46	0.112	-0.132	-0.269*
GQS	0.548**	1	0.879**	0.175	0.203	0.26	0.132	0.220	-0.216
RS	0.622**	0.879**	1	0.161	0.179	0.057	0.122	0.67	0.222

* *p* < 0.05, ** *p* < 0.01.

The inter-rater reliability ICC values varied from 0.962 to 0.972, and for the intra-rater reliability, the ICC values varied from 0.809 to 0.927, depicting almost perfect agreement.

## DISCUSSION

Many patients search the Internet for extracting information about their orthodontic treatment. YouTube™ is a media that provides rich visual content and easy access, unlike other scientific platforms that provide more academic and accurate information.^20^ However, the validity of information on YouTube^™^ is questionable, as the videos can be shared by anyone, and there is no standardization of the content for the uploaded videos. Knösel and Jung^21^ conducted a study to assess the level of knowledge related to orthodontic posts on YouTube. The authors concluded that even though YouTube™ was a platform for sharing patient experiences, the quality of the related videos was inadequate in terms of content. Singh et al^19^ investigated the quality of information on YouTube™ on rheumatoid arthritis, and they concluded that there was no standard for the quality of information available on YouTube, and there was no difference in popularity and number of views between useful and misleading videos. One of the videos from a YouTube™ channel presented yoga therapy for pain management, recommending the natural therapy for increasing the individual pain threshold. Al-Silwadi et al^6^ emphasized that visual and auditory social media sources such as YouTube™ had a positive impact on the knowledge levels of orthodontic patients. The availability of good quality of information and content on YouTube™ is essential because the feedback on these videos affects the decision-making process of the patients regarding orthodontic treatment. 

The cooperation and knowledge of orthodontic patients have an integral role in successful treatment outcomes. One of the main issues that patients and parents always consider regarding treatment is the pain associated with it. The video characteristics of this study showed that YouTube™ users viewed videos about orthodontic treatment and the pain management related to it at high rates, often uploaded videos, and frequently interacted with other users via likes, dislikes, and comment features. The variety of content available on YouTube™ regarding orthodontic pain management included videos related to wire tightening, e-chains/elastics, separator placement, band placement, Invisalign, expansion appliances and functional appliances. Only 24 (38.7%) videos were uploaded by Doctors, and those had a lesser viewing rate than 38 (61.3%) Non-doctors videos. These results were similar to the studies examining YouTube™ videos, which reported that videos uploaded by non-doctors were viewed more.^22^ ^,^ ^23^ The length of the videos uploaded by the Doctors group was shorter than the Non-doctors group, as they focused more on the topic; whereas the videos in the Non-doctors group were in the weblog format. 21 (33.8%) videos were uploaded by the patients, in which they shared their individual experiences, which was consistent with similar studies.^11^ ^,^ ^22^ ^,^ ^23^ Surprisingly, the Interaction rate was better in videos from the Doctors group. The reason behind this could be the lesser number of dislikes and lesser time elapsed since the upload of the Doctors videos. 

Lena and Dindaroglu^24^ conducted a study evaluating the content and quality of YouTube™ videos related to lingual orthodontics, and concluded that the content and quality of the videos were not adequate. Hatipoglu and Gas^25^ investigated the quality of YouTube™ videos on surgically-supported rapid palatal expansion, and showed that only 25.76% of all videos were of moderate content quality and the remaining videos had low-quality content, and no high-quality content videos were available. In this study, most videos were found to be of poor content quality (56.45%) and poor reliability (53.22%). Only 18.33% of the videos had good content quality and 11.30% of videos had good reliability, which was similar to the studies by Yagci^26^ on denture care, and Aydin MF et al^27^ and Kilinc et al^28^ on the reliability of videos on orthodontics. The categorization between Doctors and Non-doctors showed no difference in videos for the content quality, but the reliability was fairly better in the Doctors than in Non-doctors videos. These results were unlike the study by Cakmark^23^ on YouTube™ videos related to umbilical hernia. Additionally, the authors uploading the videos on YouTube™ were not evaluated by a scientific peer-review process, and were not asked for the source of information of their videos.^13^ ^,^ ^19^ ^,^ ^29^ The audio and video quality of the image was found to be substantially good, which was similar to the study by Sorenson et al.^15^

In the current study, a strong positive correlation was found between content quality and reliability, which was in contrast to the study by Ustdal et al^30^ on videos related to retainers, whereas a moderate correlation of AVQ with the content quality and reliability score was observed. Likewise, no definite correlation was found among AVQ, GQS, and RS with the number of views, likes, dislikes, and comments to the videos, similar to the study by Ustdal et al.^30^ But an indifferent result was observed in a study of YouTube™ videos on paediatric adenotonsillectomy by Sorenson et al,^15^ in which the number of likes on the videos was correlated with the quality of the video images.

## LIMITATIONS

Limitations in this study include the fact that videos were assessed for whether or not they “mentioned” a specific treatment or aetiology, not whether they advocated it. This is a major distinction, as somebody may mention a given treatment or aetiology as wrong, but the data collected does not make that distinction. In addition, even though the most commonly used keywords were searched pertaining to orthodontic pain using the Google Trends application, it should be kept in mind that different videos may be accessed using different keywords. This study generalizes orthodontic pain, which may be caused due to several reasons, such as mini-implant insertion, wire tightening, etc. Also, this study was conducted only on YouTube™ videos, and other social media sites were not evaluated. So, further study can be conducted evaluating other social media platforms.

## CONCLUSION

» According to the study, YouTube™ cannot be considered a reliable source of information in terms of quality and reliability on videos related to orthodontic pain management.
» Even though the videos uploaded by Doctors were better in terms of quality and reliability, as compared to Non-doctors videos; overall, both groups do not serve as a good source of information.
» The audio-visual quality of the uploaded videos by both Doctors and Non-doctors was adequate.
» The viewing rate was better in the Non-doctors videos, but the interaction index of the Doctors videos was better.

The videos uploaded by doctors, hospital channels, or health professionals should also be censored through an effective control mechanism. Collaboration amongst the orthodontic specialists, health professionals, patients, hospital channels, and experts in digital technology is of utmost necessity to ensure that videos related to orthodontic pain management contain relevant, high-quality, and evidence-based information. 

## References

[1] Atkinson NL, Saperstein SL, Pleis J (2009). Using the internet for health-related activities findings from a national probability sample. J Med Internet Res.

[2] Hassona Y, Taimeh D, Marahleh A, Scully C (2016). YouTube as a source of information on mouth (oral) cancer. Oral Dis.

[3] Huang CY, Shen YC, Chiang IP, Lin CS (2007). Concentration of Web users' online information behaviour.

[4] (2023). YouTube users statistics 2023.

[5] Osman W, Mohamed F, Elhassan M, Shoufan A (2022). Is YouTube a reliable source of health-related information A systematic review. BMC Med Educ.

[6] Al-Silwadi FM, Gill DS, Petrie A, Cunningham SJ (2015). Effect of social media in improving knowledge among patients having fixed appliance orthodontic treatment a single-center randomized controlled trial. Am J Orthod Dentofacial Orthop.

[7] Oliver RG, Knapman YM (1985). Attitudes to orthodontic treatment. Br J Orthod.

[8] Wu AK, McGrath C, Wong RW, Wiechmann D, Rabie AB (2010). A comparison of pain experienced by patients treated with labial and lingual orthodontic appliances. Eur J Orthod.

[9] Krishnan V, Davidovitch Z (2006). Cellular, molecular, and tissue-level reactions to orthodontic force. Am J Orthod Dentofacial Orthop.

[10] Krishnan V (2007). Orthodontic pain from causes to management--a review. Eur J Orthod.

[11] Firestone AR, Scheurer PA, Bürgin WB (1999). Patients' anticipation of pain and pain-related side effects, and their perception of pain as a result of orthodontic treatment with fixed appliances. Eur J Orthod.

[12] Hegarty E, Campbell C, Grammatopoulos E, DiBiase AT, Sherriff M, Cobourne MT (2017). YouTube(tm) as an information resource for orthognathic surgery. J Orthod.

[13] Desai T, Shariff A, Dhingra V, Minhas D, Eure M, Kats M (2013). Is content really king An objective analysis of the public's response to medical videos on YouTube. PLoS One.

[14] Blandford A (2015). Google, public libraries, and the deep web. DJIM.

[15] Sorensen JA, Pusz MD, Brietzke SE (2014). YouTube as an information source for pediatric adenotonsillectomy and ear tube surgery. Int J Pediatr Otorhinolaryngol.

[16] Bernard A, Langille M, Hughes S, Rose C, Leddin D, Veldhuyzen van Zanten S (2007). A systematic review of patient inflammatory bowel disease information resources on the World Wide Web. Am J Gastroenterol.

[17] Li M, Yan S, Yang D, Li B, Cui W (2019). YouTube(tm) as a source of information on food poisoning. BMC Public Health.

[18] Charnock D, Shepperd S, Needham G, Gann R (1999). DISCERN an instrument for judging the quality of written consumer health information on treatment choices. J Epidemiol Community Health.

[19] Singh AG, Singh S, Singh PP (2012). YouTube for information on rheumatoid arthritis--a wakeup call. J Rheumatol.

[20] Fox S, Duggan M (2013). 35% of U.S. adults have gone online to figure out a medical condition; of these, half followed up with a visit to a medical professional.

[21] Knösel M, Jung K (2011). Informational value and bias of videos related to orthodontics screened on a video-sharing Web site. Angle Orthod.

[22] Cetin A (2021). Evaluation of YouTube video content related to the management of hypoglycemia. Cureus.

[23] Cakmak G (2021). Evaluation of scientific quality of YouTube video content related to umbilical hernia. Cureus.

[24] Lena Y, Dindaroglu F (2018). Lingual orthodontic treatment a YouTube(tm) video analysis. Angle Orthod.

[25] Hatipoglu S, Gas S (2020). Is Information for surgically assisted rapid palatal expansion available on YouTube reliable. J Oral Maxillofac Surg.

[26] Yagci F (2023). Evaluation of YouTube as an information source for denture care. J Prosthet Dent.

[27] Aydin MF, Aydin MA (2020). Quality and reliability of information available on YouTube and Google pertaining gastroesophageal reflux disease. Int J Med Inform.

[28] Kilinç DD, Sayar G (2019). Assessment of reliability of YouTube videos on orthodontics. Turk J Orthod.

[29] Steinberg PL, Wason S, Stern JM, Deters L, Kowal B, Seigne J (2010). YouTube as source of prostate cancer information. Urology.

[30] Ustdal G, Guney AU (2020). YouTube as a source of information about orthodontic clear aligners. Angle Orthod.

